# A New Look at Trigger Point Injections

**DOI:** 10.1155/2012/492452

**Published:** 2011-09-29

**Authors:** Clara S. M. Wong, Steven H. S. Wong

**Affiliations:** Department of Anaesthesiology, Queen Elizabeth Hospital, 30 Gascoigne Road, Kowloon, Hong Kong

## Abstract

Trigger point injections are commonly practised pain interventional techniques. However, there is still lack of objective diagnostic criteria for trigger points. The mechanisms of action of trigger point injection remain obscure and its efficacy remains heterogeneous. The advent of ultrasound technology in the noninvasive real-time imaging of soft tissues sheds new light on visualization of trigger points, explaining the effect of trigger point injection by blockade of peripheral nerves, and minimizing the complications of blind injection.

## 1. Introduction

Myofascial pain syndrome is a common, painful musculoskeletal disorder characterized by the presence of trigger points. They have been implicated in patients with headache, neck pain, low back pain, and various other musculoskeletal and systemic disorders [1–4]. The prevalence of myofascial trigger points among patients complaining of pain anywhere in the body ranged from 30% to 93% [5]. Although the most important strategy in treatment of myofascial pain syndrome is to identify the etiological lesion that causes the activation of trigger points and to treat the underlying pathology [6], trigger point injections are still commonly practised pain interventional technique for symptomatic relief. 

Despite the popularity of trigger point injections, the pathophysiology of myofascial trigger points remains unclear. Localization of a trigger point is often based on the physician's examination. However, such physical examination is often unreliable. Lack of objective clinical measurements has also been a barrier for critically evaluating the efficacy of the therapeutic methods.

Ultrasound is used extensively for noninvasive real-time imaging of soft tissues including muscle, nerve, tendon, fascia, and blood vessels. With the advent of portable ultrasound technology, ultrasound is now commonly employed in the field of regional analgesia. In this paper, we will look at the potential application of ultrasound in trigger point injections.

## 2. Diagnosis of Trigger Points

Physician's sense of feel and patient expressions of pain upon palpation are the most commonly used method to localize a trigger point. The most common physical finding is palpation of a hypersensitive bundle or nodule of muscle fibre of harder than normal consistency. The palpation will elicit pain over the palpated muscle and/or cause radiation of pain towards the zone of reference in addition to a twitch response [7].

In myofascial pain syndrome, trigger points have been classified into active or latent. In an active trigger point, there is an area of tenderness at rest or on palpation, a taut band of muscle, a local twitch response, and referred pain elicited by firm compression similar to the patient's complaint. Latent trigger points are more commonly seen. They may display hypersensitivity and exhibit all the characteristics of an active trigger point except that it is not associated with spontaneous pain [7]. 

Trigger points have also been further classified into key or satellite. An active key trigger point in one muscle can induce an active satellite trigger point in another muscle. Inactivation of the key trigger point often also inactivates its satellite trigger point without treatment of the satellite trigger point itself [7]. 

The diagnosis of trigger points depends very much on the subjective experience of the physician. Pressure algometry has been used to quantify the sensitivity of trigger points. A hand-held pressure meter with a 1 cm^2^ rubber disc attached to a force gauge calibrated up to 10 kg is applied over a trigger point to measure its pain threshold [8]. However, this method is not commonly employed clinically, and there have not been any imaging criteria for the diagnosis of trigger points.

## 3. Pathophysiology of Trigger Points

Trigger points are defined as palpable, tense bands of skeletal muscle fibres. They can produce both local and referred pain when compressed. 

The local pain could be explained by the tissue ischemia resulting from prolonged muscle contraction with accumulation of acids and chemicals such as serotonin, histamine, kinins, and prostaglandins [9]. These changes are fed into a cycle of increasing motor or sympathetic activity and can lead to increased pain. A painful event can sustain itself once a cycle is established even after the initial stimulus has been removed [10]. 

The pathogenesis of trigger points is probably related to sensitized sensory nerve fibres (nociceptors) associated with dysfunctional endplates [11]. In fact, endplate noise was found to be significantly more prevalent in myofascial trigger points than in sites that were outside of a trigger point but still within the endplate zone [12]. 

Studies have found that development of trigger points is dependent on an integrative mechanism in the spinal cord. When the input from nociceptors in an original receptive field persists (pain from an active trigger point), central sensitization in the spinal cord may develop, and the receptive field corresponding to the original dorsal horn neuron may be expanded (referred pain). Through this mechanism, new “satellite trigger points” may develop in the referred zone of the original trigger point [11]. 

## 4. Mechanisms of Action of Trigger Point Injections

Noninvasive measures for treatment of trigger points include spray and stretch, transcutaneous electrical stimulation, physical therapy, and massage. Invasive treatments include injections with local anaesthetics, corticosteroids, or botulinum toxin, or dry needling [13–18]. 

Hong reported that with either lidocaine injection or dry needling of trigger points, the patients experienced almost complete relief of pain immediately after injection if local twitch responses were elicited. On the other hand, they experienced only minimal relief if no such response occurred during injection. Hong has suggested that nociceptors (free nerve endings) are encountered and blocked during trigger point injection if local twitch response can be elicited [19]. 

The mechanism of action of trigger point injections is thought to be disruption of the trigger points by the mechanical effect of the needle or the chemical effect of the agents injected, resulting in relaxation and lengthening of the muscle fibre. The effect of the injectate may include local vasodilation, dilution, and removal of the accumulated nociceptive substrates. Botulinum toxin A has been used to block acetylcholine release from the motor nerve ending and subsequently relieve the taut band [6].

While the relief of local pain could easily be explained by the relaxation of the muscle fibre, the relief of referred pain could not be explained without attributing it to a peripheral nerve blockade. However, little has been said in the literature regarding the mechanism of trigger point injection in this respect. 

## 5. Could the Application of Ultrasound Solve the Mystery of Trigger Points?

### 5.1. Direct Visualization of Trigger Points

As mentioned above, the most common physical finding of a trigger point is palpation of a hypersensitive bundle or nodule of muscle fibre of harder than normal consistency. Attempts to confirm the presence of myofascial trigger points using imaging have been demonstrated by magnetic resonance elastography [20]. For ultrasound, earlier studies have failed to find any correlation between physical findings and diagnostic ultrasound [21]. This may be attributed to poorer quality of ultrasound imaging in earlier dates. 

Recently, Sikdar et al. have tried to use ultrasound to visualize and characterize trigger points. They found that trigger points appeared as focal, hypoechoic regions of elliptical shape, with a size of 0.16 cm [22]. This is promising as ultrasound can provide a more objective diagnosis of trigger point. Even if visualization of individual trigger point is difficult due to the small size, some advocate the use of ultrasound to guide proper needle placement in muscle tissue and to avoid adipose or nonmusculature structures during trigger point injections [23]. 

### 5.2. Injection of Peripheral Nerves

Trigger point injections have been implicated in patients with headache, low back pain, and various other musculoskeletal and systemic disorders. Some of these injections may involve injectate deposition directly to the nerves supplying the region. Indeed, entrapment, compression, or irritation of the sensory nerves of local regions has been implicated in various conditions.

#### 5.2.1. Greater Occipital Nerve

Entrapment of the greater occipital nerve is often implicated as the cause of cervicogenic headache, and the characteristic occipital headache can be reproduced by finger pressure over the corresponding occipital nerve over the occipital ridge [3, 24–26]. This referral pattern of pain coincides with that of the properties of a trigger point, and it could explain the mechanism of referred pain for trigger points.

Simons has considered that the effect of greater occipital nerve injection is due to the release of the entrapment by relaxation of semispinalis muscle [7]. However, injection of local anaesthetics with or without steroid over the occipital nerve has been found to result in alleviation of occipital headache [27]. In migraine headaches, local injection of local anaesthetics or botulinum toxin type A to the greater occipital nerve has been demonstrated to provide relief of the condition [24]. 

There are several techniques of ultrasound-guided blockade of greater occipital nerve. The classical distal block technique involves placing the transducer at the superior nuchal line, while for the new proximal approach, the transducer is placed at the level of C2, and the greater occipital nerve lies superficial to the obliquus capitis inferior muscle [28, 29]. 

#### 5.2.2. Abdominal Cutaneous Nerve

Kuan et al. showed that local injection of anaesthetics or steroid can treat some patients with lower abdominal pain presenting with trigger points in the abdomen, thus avoiding diagnostic laparoscopy and medications [30]. 

Trigger points over the abdominal wall may in fact be entrapped cutaneous nerves. Peripheral nerve entrapment (e.g., ilioinguinal-iliohypogastric nerves, thoracic lateral cutaneous nerve) has been suggested to cause lower abdominal pain [31, 32]. 

Ultrasound-guided blocks for ilioinguinal and iliohypogastric nerves have been practised widely in anaesthesia [33–35]. Recently, ultrasound-guided transversus abdominis plane (TAP) block is also commonly used to provide postoperative pain relief for patients undergoing laparotomy [35–38]. 

By placing the ultrasound probe about 5 cm cranial to the anterior superior iliac spine, the ilioinguinal and iliohypogastric nerves can be found between the transverse abdominal and the internal oblique muscle [39]. For TAP block, the transducer can be placed in a transverse plane between the iliac crest and the anterior axillary line. Local anaesthetics can be deposited between the transversus abdominis muscle and the internal oblique muscle [40]. 

#### 5.2.3. Dorsal Ramus of Spinal Nerve

Low back pain is a common chronic pain syndrome; however, in most cases, a specific diagnosis cannot be established. Trigger point injections have been found to relieve myofascial low back pains. However, there has been lack of evidence in the literature to support its efficacy. This could be attributed to the heterogeneity in the diagnosis and technique of localization of trigger points in low back pain. Most of the studies employed subjective localization of trigger points, and the techniques of localization and injection of trigger points were not well described. 

Miyakoshi et al. demonstrated that CT-guided total dorsal ramus block was effective in the treatment of chronic low back pain in a group of patients with overlapping facet syndrome with myofascial syndrome with pain originating from myofascial structure, facet joint, or both [41]. They demonstrated that a single injection of a larger volume of local anaesthetics over the conventional target point for medial branch block, which was the junction of the L5 superior articular process and the transverse process, was effective to block the medial, intermediate, and lateral branches of the lumbar dorsal ramus, with significantly better pain reduction compared to conventional trigger point injection. The findings in this study shed light to the possibility of relief of myofascial pain syndrome by a single nerve injection. It may explain the poor results of pure intramuscular injections in controlled studies, in contrast to the better results with uncontrolled studies and case reports, in which some of the results may be attributable to accidental nerve injection using the conventional blind injection techniques.

For ultrasound-guided medial branch block, the transducer is first placed longitudinally to find the respective transverse process and localize the lumbar level. Then the transducer can be rotated into a transverse plane to delineate the transverse process and the superior articular process of the adjacent facet joint. The bottom of the groove between the lateral surface of the superior articular process and the cephalad margin of the respective transverse process was defined as the target site [42]. 

Ultrasound-guided technique may be adapted to perform injection of the lower back, targeting at the dorsal rami of the lumbar spinal nerves to increase the efficacy of injection.

#### 5.2.4. Lumbar Plexus

There have been case reports on the use of trigger point injection for treatment of pain that was remote from the site of trigger points. Interestingly, Iguchi et al. used trigger point injection for the amelioration of renal colic. In their paper, they described the injection technique as follows. Trigger points were located over the paraspinal region at around L3 level. A long needle (23-gauge 6 cm) was inserted deep into the trigger points, and 5–10 mL of 1% lignocaine was injected [43]. Such injection was in fact into the psoas muscle, and the effect could be attributed to a lumbar plexus block. 

Lumbar plexus block with ultrasound guidance has been described. A curved transducer can be placed in the transverse plane at L2–L4 level for the lumbar plexus block. This transverse view should show the psoas muscle without the transverse process. The target of the needle tip is within the posterior 1/3 of the psoas muscle bulk [40]. 

#### 5.2.5. Pudendal Nerve

Langford et al. reported the effective use of levator ani trigger point injection in the treatment of chronic pelvic pain. Trigger points were identified by manual intravaginal palpation, and the trigger points were injected with a large volume (up to about 20 mL) of a mixture of local anesthetics and depot steroid. The effect of such injection might in fact be caused by the concomitant pudendal nerve block [44]. 

Pudendal nerve blockade with ultrasound guidance can be performed via the transgluteal approach. The probe is placed transverse to the posterior superior iliac spine and moved caudally until the piriformis muscle is seen. The probe is then moved further caudad to identify the ischial spine, in which the pudendal nerve will be seen lying medial to the pudendal artery [29]. 

## 6. Other Advantages of Ultrasound in Trigger Point Injections

Trigger point injections are commonly performed in clinics as an outpatient procedure. Serious complications, although of rare occurrence, have been reported (e.g., pneumothorax, haematoma, intravascular injection of local anaesthetics, and intrathecal injections) [45]. Direct visualization of surrounding soft tissues and important structures can reduce the risk of such complications. Moreover, ultrasound allows real-time imaging of the spread of the injectate around the relevant structures and increases the success rate of injection.

## 7. Future Directions

The nonspecific diagnosis and lack of objective clinical measurements for trigger points mean that the evidence for the effectiveness of trigger point injection remains heterogenous. There is so far no strong evidence for the effectiveness of trigger point injections, and many physicians consider trigger point injections a little more than, if not equivalent to, placebo effects.

With the advancement of ultrasound technology, the quality of scans for soft tissues and musculature has improved dramatically. Future studies may focus on more objective diagnostic criteria of trigger points using ultrasound imaging. For the technique of trigger point injections, real-time visualization of trigger points, relaxation of locally contracting muscles, and visualization of surrounding tissues or important structures may improve the outcome and minimize complications of such treatments. 

Moreover, efficacy of some of the trigger point injections traditionally performed may be related to some kind of peripheral nerve blocks, the implication which is yet to be explored. 
